# 

**Published:** 1911-06

**Authors:** 


### BOOKS RECEIVED

Medical Jurisprudence, Forensic Medicine and Toxicology. By R. A. Witthaus, A.M., M.D., Professor of Chemistry, Toxicology and Medical Jurisprudence in Cornell University, and Tracy C. Becker, A.B., LL.D., Counsellor at Law, Professor of Criminal Law and Medical Jurisprudence in the University of Buffalo. Second edition. Vol. IV. Octavo, pp 1271. Illustrated. New York: William Wood & Co. 1911. (Muslin, $24.00 for set; singly, $7.00 each. Half morocco, $28.00 for set; singly, $8.00 each.)

Food and the Principles of Dietetics. By Robert Hutchinson, M.D., Edin., F.R.C.P., Physician to the London Hospital; Physician with charge of Out-Patients to the Hospital for Sick Children, Great Ormond Street. Third edition. Octavo, pp. 615. With plates and diagrams. New York: William Wood & Co. 1911. (Cloth, $3.00.)

An Introduction to Dermatology. By Norman Walker, M.D., F.R.C.P., Physician for Diseases of the Skin, the Royal Infirmary, Edinburgh. Fifth edition. Octavo, pp. 346. With 43 colored plates and 79 illustrations. New York: William Wood & Co. 1911. (Cloth, $4.00.)

The Practical Medicine Series. Ten Volumes. Under the general editorial charge of Gustavus P. Head, M.D. Vol. II. General Surgery. Edited by John B. Murphy, A.M., M.D., LL.D., Professor of Surgery in Northwestern University. Series 1911. Chicago: The Year Book Publishers. (Price, $2.00; entire series, $10.00.)

Plaster of Paris and How to Use it. By Martin W. Ware, M.D., N. Y., Adjunct Attending Surgeon, Mount Sinai Hospital; Surgeon to the Good Samaritan Dispensary; Instructor of Surgery in the New York Post-Graduate Medical School. Second edition revised and enlarged. New York: Surgery Publishing Co. (Cloth, $1.25.)

Gonorrhea in the Male. A Practical Guide to its Treatment. By Abr. L. Wolbarst, M.D., Consulting Genitourinary Surgeon, Central

Islip State Hospital. New York: International Journal of Surgery Co. 1911. (Cloth, $1.00.)

Report of the Commissioner of Education. For the year ended June 30, 1910. Vol. II. Washington: Government Printing Office.

Report from Pathological Department, Central Indiana Hospital for the Insane. Vol. II, 1906-07, 1907-08. Vol. III, 1908-1909.

Health Hints. By E. R. Pritchard, Secretary of the Chicago Department of Health. Small 12 mo, pp. 153. Chicago: The Reilly & Britton Co. (Cloth, 50 cents.)

1,000 Surgical Suggestions. By Walter M. Brickner, B.S., M.D., Adjunct Surgeon Mount Sinai Hospital, Editor in Chief American Journal of Surgery. Fourth edition. 12 mo, pp. 225. New York: Surgery Publishing Co. (Cloth, $1.00.)

Transactions of the American Association of Genitourinary Surgeons. Twenty-third Annual Meeting held at Washington, D. C., May 3-5, 1910. Vol. V. Edward L. Keyes, Jr., M.D., Secretary.

Tuberculosis, as a Disease of the Masses, and How to Combat It. By S. A. Knopf, M.D. Awarded the International Prize by the Committee of the International Congress to Combat Tuberculosis as a Disease of the Masses, which convened at Berlin, May 24-27, 1909. Seventh edition. New York: The Survey; also for sale by F. P. Flori, New York. 1911. (Paper, 25 cents.)

What to Eat and Why. By G. Carroll Smith, M.D., of Boston, Mass. Octavo of 310 pages. Philadelphia and London: W. B. Saunders Company. 1911. (Cloth, $2.50 net.)

A Textbook of Medical Diagnosis. By James M. Anders, M.D., Professor of the Theory and Practice of Medicine and of Clinical Medicine, and L. Napoleon Boston, M.D., Adjunct Professor of Medicine, Medico-Chirurgical College, Philadelphia. Octavo of 1,195 pages, with 443 illustrations, 17 in colors. Philadelphia and London: W. B. Saunders Company. 1911. (Cloth, $6.00 net; half morocco, $7.50 net.)

Practical Cystoscopy and the Diagnosis of Surgical Diseases of the Kidneys and Urinary Bladder. By Paul M. Pilcher, M.D., Consulting Surgeon to the Eastern Long Island Hospital. Octavo of 398 pages, with 233 illustrations, 29 in colors. Philadelphia and London: W. B. Saunders Company. 1911. (Cloth, $5.50 net.)

A Handbook of Practical Treatment. In three volumes. By 79 eminent specialists. Edited by John H. Musser, M.D., Professor of Clinical Medicine, University of Pennsylvania; and A. O. J. Kelly, M.D., Assistant Professor of Medicine, University of Pennsylvania. Volume II. Octavo of 865 pages. Illustrated. Philadelphia and London: W. B. Saunders Company. 1911. (Per volume: Cloth, $6.00 net; half morocco, $7.50 net).

